# Generalized Parameter-Adjusted Stochastic Resonance of Duffing Oscillator and Its Application to Weak-Signal Detection

**DOI:** 10.3390/s150921327

**Published:** 2015-08-28

**Authors:** Zhi-Hui Lai, Yong-Gang Leng

**Affiliations:** 1 School of Mechatronics Engineering, Nanchang University, Nanchang 330031, China; 2 School of Mechanical Engineering, Tianjin University, Tianjin 300072, China; E-Mail: leng_yg@tju.edu.cn

**Keywords:** Duffing oscillator, stochastic resonance, Kramers rate, generalized parameter-adjustment, weak-signal detection

## Abstract

A two-dimensional Duffing oscillator which can produce stochastic resonance (SR) is studied in this paper. We introduce its SR mechanism and present a generalized parameter-adjusted SR (GPASR) model of this oscillator for the necessity of parameter adjustments. The Kramers rate is chosen as the theoretical basis to establish a judgmental function for judging the occurrence of SR in this model; and to analyze and summarize the parameter-adjusted rules under unmatched signal amplitude, frequency, and/or noise-intensity. Furthermore, we propose the weak-signal detection approach based on this GPASR model. Finally, we employ two practical examples to demonstrate the feasibility of the proposed approach in practical engineering application.

## 1. Introduction

The term Stochastic Resonance (SR) was first coined by Benzi *et al.*, and used to explain the switching of the Earth’s climate between ice ages and periods of relative warmth over a roughly 100,000-year cycle [1,2,3]. Subsequently, SR phenomena have been observed in Schmitt trigger circuit by Fauve [4] and a bidirectional ring laser by McNamara [5], which confirmed the SR theory. Since then, this nonlinear phenomenon has received much attention from the physics community, which was widely used in weak-signal detection and energy harvesting [6,7,8,9]. Recent researches show that SR not only appears in bistable systems [10], but also in monostable oscillators [11], chaotic systems [12], and time-delay systems [13].

The essential ingredients for SR consist of a nonlinear system, a weak signal, and a source of noise. Using the nonlinear system, the output signal-to-noise ratio (SNR) of the system will peak at a certain value of noise intensity with the synergistic action of the input signal and noise. This is similar to the well-known resonance phenomenon in mechanics and why SR was so-called. When SR occurs, a certain fraction of the noise energy is transferred to a weak signal and greatly strengthens its intensity. Therefore, many studies have exploited SR for weak-signal detection and achieved positive research results [14,15,16,17,18].

The system of two-dimensional Duffing oscillator can also produce SR. Gammaitoni *et al*. first introduced SR for Duffing oscillator [19,20], and its output characteristics were studied theoretically and in simulations [21,22]; the nonlinear phenomena were also observed in circuit experiments [23], and [24,25] presented a detection model based on Duffing oscillator to realize weak-signal detection.

SR describes an optimal match between signal, noise and nonlinear system. However, the characters of signal and noise are always unknown in practical engineering application and do not always match with the system. Thus, one, two or even all character(s) of signal, noise and system must be adjusted to realize weak-signal detection by using Duffing oscillator in the context of SR. For the measured signal, the only feasible way is to adjust system parameters to realize the optimal match between signal, noise and system. The parameter-adjusted SR of one-dimensional Langevin system has been sufficiently studied [26,27,28], however, only few researches began to focus on the parameter-adjusted SR in Duffing system. In recent research [29,30] the influence of SR in Duffing oscillator against damping ratio was studied, and [24] introduced a scale-transformation coefficient to realize SR for a large-frequency input signal. These research results lay the foundation for further studies on parameter-adjusted SR in a Duffing oscillator. However, there exist following shortcomings: (i) Current research focuses on simulation analysis, but rarely studies the parameter-adjusted SR mechanism; (ii) Most research focuses on studying parameter-adjusted rules of one parameter, but rarely studies the relevance between parameters; (iii) Little research is concerned with its application in engineering practice such as weak-signal detection; the effectiveness of the detection method based on Duffing SR has not been verified and the applicability has not been studied.

The mentioned shortcomings of current research restrict the application of parameter-adjusted SR of a Duffing oscillator, so there remain many topics worth exploring. We need to find a theoretical tool to systematically analyze the parameter-adjusted SR mechanism of single parameters and the relevance between parameters, to thus obtain the parameter-adjusted rules for a Duffing oscillator to produce SR and finally propose a relevant method to realize the weak-signal detection based on parameter-adjusted SR of a Duffing oscillator. In our preliminary research [31], a model of generalized parameter-adjusted SR (GPASR) was presented and we systematically studied the parameter-adjusted rules for the Duffing system to better produce SR. However, the adjusted rules summarized in [31] were not complete because we did not fully discuss the situations that the input signal amplitude does not match with other parameters, and we also need to propose a GPASR-based weak-signal detection method and realize its application on engineering practice such as incipient fault diagnosis of mechanical equipment.

Therefore, the GPASR in a Duffing oscillator is further investigated and developed in this paper. Section 2 introduces the principle of SR in a Duffing oscillator. We present the generalized parameter-adjusted model of a Duffing oscillator and classify parameters in Section 3; we also analyze the parameters of a Duffing system based on Kramers rate, and comprehensively study the mechanism of GPASR in a Duffing oscillator when the signal amplitude, frequency and/or noise-intensity are unmatched in this section. Section 4 presents the weak-signal detection method based on the GPASR of a Duffing oscillator, along with two practical examples. In Section 5, we provide a summary.

## 2. Principle of SR in a Duffing Oscillator

The SR model of a two-dimensional Duffing oscillator can be described as [32]:

(1) $$\ddot{x}+k\dot{x}-ax+bx^{3}=A\text{cos}(2\text{π}f_{0}t)+\sqrt{2D}\text{ξ}(t)$$

where *k* denotes the damping ratio; $-ax+bx^{3}$ is the force of the potential field, whose potential function $U(x)=-ax^{2}/2+bx^{4}/4$, *a* and *b* are positive system parameters; $s(t)=A\text{cos}(2\pi f_{0}t)$ describes a harmonic signal with amplitude *A*, frequency *f*_0_ and zero initial phase; $n(t)=\sqrt{2D}\text{ξ}(t)$ is the noise, where *D* is the intensity and ξ(t) represents Gaussian white noise with zero-mean and unit-variance. Thus, Equation (1) is representative of a Duffing oscillator simultaneously driven by a characteristic signal s(t) and noise n(t). 

**Figure 1:**
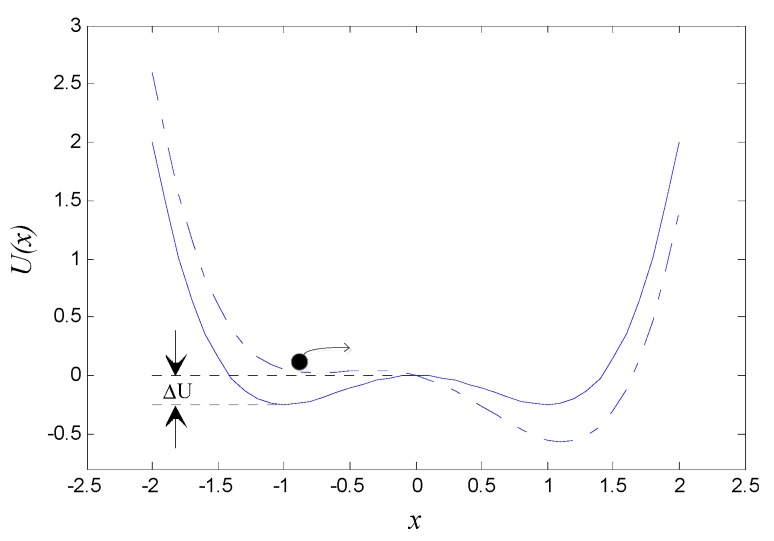
Bistable potential function of the Duffing system without driving force (solid line) and potential changes with driving force (dotted line). Switching events may take place in the presence of noise as indicated by the arrow.

The output x(t) of Equation (1) can be understood as the trajectory of a unit-mass Brownian particle moving in the potential field U(x) under the coaction of damping force $-k\dot{x}$, potential field force −dU(x)/dx, periodic driving force s(t) and random noise n(t), as shown in Figure 1.

It can be seen from Figure 1 that, in the absence of both characteristic signal and noise, *i.e.*, A=0 and D=0, the potential function describes a bistable potential field with two stable equilibrium points at $x_{m1,m2}=\pm \sqrt{a/b}$ and one unstable equilibrium point at $x_{b}=0$. The height of the potential barrier $\Delta U=a^{2}/(4b)$. However, when a periodic signal is present while noise is absent, namely A≠0 and D=0, the potential function is modulated periodically by the characteristic signal and transforms from U(x) to V(x):

(2) $$V(x)=U(x)-xA\text{cos}(2\text{π}f_{0}t)=-ax^{2}/2+bx^{4}/4-xA\text{cos}(2\text{π}f_{0}t)$$

Hence, the potential wells of the potential function will alternately raise or fall periodically; see Figure 1. There exists for the bistable system a critical amplitude, whose value $A_{C}=\sqrt{4a^{3}/(27b)}$ [33]. When $A<A_{C}$, the barrier still remains between the potential wells inhibiting the Brownian particle in jumping freely to the adjacent well. Therefore the particle can only oscillate in one well; while when $A>A_{C}$, the Brownian particle can pass over the barrier and oscillate between two wells.

Interestingly, with the injection of noise at the input, namely D≠0, and all the parameters are appropriate, even though $A<A_{C}$, the Brownian particle can accumulate enough energy to cross the potential barrier with the assistance of noise, also see Figure 1. Thus noise produces a positive effect on the signal when its intensity is appropriate for the signal, noise, and Duffing system to achieve synergy. A fraction of the noise energy is transferred to the signal, thereby greatly strengthening the intensity of the weak signal so that the output SNR of system will be maximized and SR appears. The noise-driven Brownian particle transits at a certain rate, which is given by the well-known Kramers rate [34]:

(3) $$r_{K}=\frac{a}{\sqrt{2}\text{π}k}\text{exp}\left(-\frac{a^{2}}{4bD}\right)$$

When the average waiting time $T_{K}(D)=1/r_{K}$ between two noise-induced interwell transitions is comparable with the changing period of the potential function (*i.e*., half the period $T=1/f_{0}$ of the periodic force), namely $T_{K}(D)=T/2$ or:

(4) $$\frac{a}{\sqrt{2}\text{π}k}\text{exp}\left(-\frac{a^{2}}{4bD}\right)=2f_{0}$$

the frequency of output signal is equal to that of input signal and the amplitude of output signal greatly strengthens, Equation (1) produces SR [34]. This is the SR mechanism of a Duffing oscillator.

**Figure 2:**
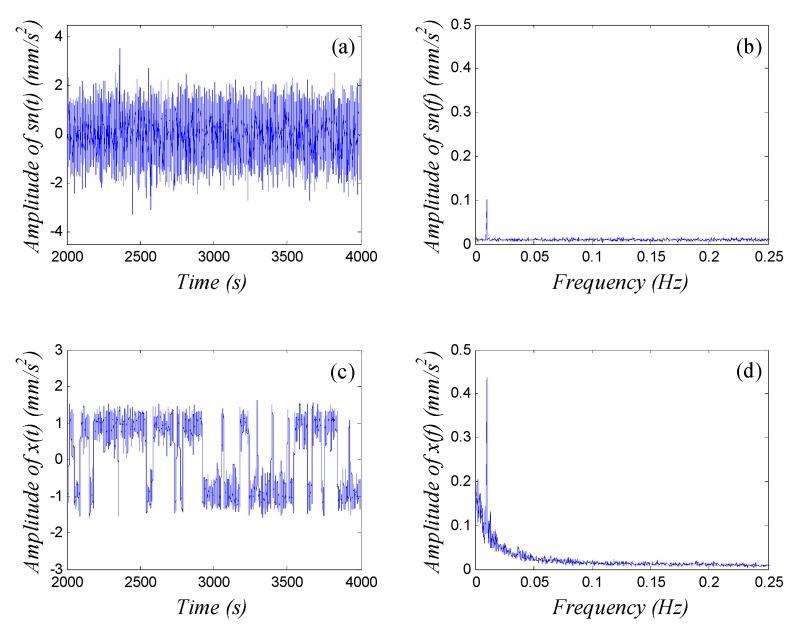
SR of the bistable Duffing equation. (**a**) waveform of input signal; (**b**) spectrum of input signal; (**c**) waveform of output signal; (**d**)spectrum of output signal.

**Figure 3:**
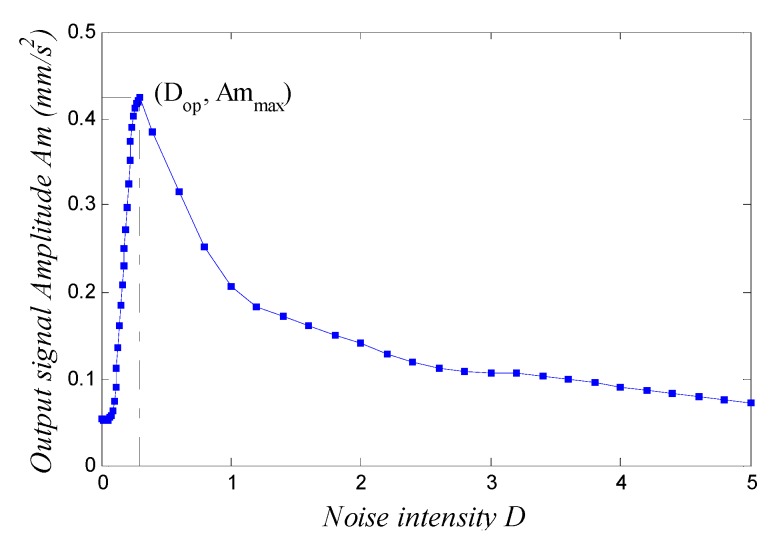
Response curve of the output signal amplitude against noise intensity.

A group of classical parameters are given below to illustrate the SR phenomenon in a Duffing system. In Equation (1), let:

(5) $$k=0.5,\;a=b=1,\;A=0.1,\;f_{0}=0.01\text{ Hz},\;D=0.29$$

the SNR of the input signal under such parameters is SNR=−20.6 dB. Here, SNR is defined as $\text{SNR}=10{\text{log}}_{10}(\psi_{s}^{2}/\psi_{n}^{2})$, where $\psi_{s}^{2}$ and $\psi_{n}^{2}$ denote the power of the signal and noise, respectively. The fourth-order Runge-Kutta algorithm is adopted to solve the differential equations unless otherwise specified. Here, we define sn(t)=s(t)+n(t) as input signal, and we set the sampling frequency $f_{s}=5$ Hz. The corresponding calculating step $h=1/f_{s}=0.2$ s, and calculating length N=20000. The spectrum is averaged over 10 cycles. Thus, the input/output waveform and spectrum of Equation (1) can be obtained, as shown in Figure 2. We see in Figure 2 that the Duffing system achieves SR under certain parameter conditions. That is to say, the output signal amplitude at $f=f_{0}$ in the output spectrum, which is denoted as Am in this paper, attains its maximum value. It is much larger than the input signal amplitude. We can plot the response curve of the output signal amplitude Am against noise intensity D among range [0,5] while maintaining other parameters constant; see Figure 3. Figure 3 shows that Am first increases and then falls off as noise-intensity increases; the peak is at a certain value $D_{op}$ (as $D_{op}=0.29$ in Figure 2). That is the typical characteristic of SR, and $D_{op}$ is the optimal noise-intensity under a group of certain parameters.

## 3. GPASR in a Duffing Oscillator Based on Kramers Rate

### 3.1. GPASR of a Duffing Oscillator

The above analysis implies that the energy in the output characteristic signal of the Duffing system, which can be manifested through the output spectrum, will strengthen when SR takes place. The Duffing system acts as a weak-signal detector to extract the weak-signal characteristics under strong noise backgrounds. However, SR requires an optimal match between signal, noise and system, which is difficult to be realized in signal detection in practical engineering. Therefore, parameter-adjusted SR in a Duffing oscillator is necessary to be studied through analyzing the influence of the realization of SR against different parameters.

There are two significances in studying the parameter-adjusted SR in a Duffing oscillator. Firstly, the adiabatic approximation theory in studying SR requires $A<A_{C}$, D≪1, and $f_{0}\ll 1$ [35]. These small-parameter limits are extremely rigorous. As is well known that the parameters of actual engineering signals seldom satisfy these limits, so the detection of these large-parameter signals can be realized through the parameter-adjusted SR. Secondly, when the signal, noise and system are unmatched, the system can also produce SR by adjusting one or more parameters. 

To realize the SR in a Duffing oscillator under large-amplitude and/or large-frequency conditions and identify the signals in practical engineering, we usually first transform the amplitude and time/frequency scale of the test signals, thus Equation (1) can be rewritten as:

(6) $$\ddot{x}+k\dot{x}-ax+bx^{3}=\text{ε}\left[A\text{cos}(2\text{π}\frac{f_{0}}{R}t^{\prime })+\sqrt{2D}\text{ξ}(t^{\prime })\right]=\text{ε}A\text{cos}(2\text{π}\frac{f_{0}}{R}t^{\prime })+\sqrt{2\varepsilon^{2}D}\text{ξ}(t^{\prime })$$

where ε is amplitude-transformation coefficient for linearly magnifying or diminishing the test signal; R is scale-transformation coefficient for transforming the time/frequency scale of test signal; $t^{\prime }=Rt$ is the time scale after scale transformation; $x=x(t^{\prime })$ is the system output in terms of scale $t^{\prime }$. Thereby, the transformation of amplitude and scale can be regarded as generalized parameter adjustments. Thus, the SR of a Duffing Equation (6) obtained by adjusting the parameters of the system is the so-called GPASR of Duffing oscillator, and Equation (6) is called generalized parameter-adjusted Duffing equation. These parameters include: damping ratio k; system parameters a and b; signal parameters A, $f_{0}$ and D; generalized parameters ε and R. All values are positive.

### 3.2. Parameters Analysis of a Duffing System Based on Kramers Rate

The parameter conditions for Duffing Equation (1) to produce SR is given by Equation (4), so we can use Kramers rate to study the mechanism of GPASR for a Duffing oscillator as well. The corresponding parameter conditions for the Duffing Equation (6) to produce SR is:

(7) $$\frac{a}{\sqrt{2}\text{π}k}\text{exp}\left(-\frac{a^{2}}{4b{\text{ε}}^{2}D}\right)=2f_{0}/R$$

Based on Equation (7), we can analyze each parameter in Equation (6). By defining Function:

(8) $$F(k,a,b,A,f_{0},D,\text{ε},R)=\frac{aR}{2\sqrt{2}\text{π}kf_{0}}\text{exp}\left(-\frac{a^{2}}{4b{\text{ε}}^{2}D}\right)$$

we can easily know that SR occurs when F=1 thus Equation (8) is called the judgmental Function to judge the occurrence of SR in Equation (6). Rules can be obtained from Equation (8):

(i) the value of F is independent of A ;
(ii) F is a monotone increasing Function of b, D, ε, and R ;
(iii) F is a monotone decreasing Function of k, and $f_{0}$;
(iv) F is a monotone increasing Function of a when $a<\text{ε}\sqrt{2bD}$, and is a monotone decreasing Function of a when $a>\text{ε}\sqrt{2bD}$.

The rules (i)–(iii) are obvious, so only a simple derivation for rule (iv) is given below. We take a derivative for Equation (8) with respect to parameter a and obtain:

(9) $$\begin{array}{c} \frac{\text{d}F}{\text{d}a}=\frac{R}{2\sqrt{2}\text{π}kf_{0}}\text{exp}\left(-\frac{a^{2}}{4b{\text{ε}}^{2}D}\right)+\frac{aR}{2\sqrt{2}\text{π}kf_{0}}\text{exp}\left(-\frac{a^{2}}{4b{\text{ε}}^{2}D}\right)\cdot \left(-\frac{2a}{4b{\text{ε}}^{2}D}\right)\\ =\frac{R}{2\sqrt{2}\text{π}kf_{0}}\text{exp}\left(-\frac{a^{2}}{4b{\text{ε}}^{2}D}\right)\left(\frac{4b{\text{ε}}^{2}D-2a^{2}}{4b{\text{ε}}^{2}D}\right) \end{array}$$

We know from Equation (9) that when dF/da>0, *i.e.*, $a<\text{ε}\sqrt{2bD}$, F is a monotone increasing Function of a; and when dF/da<0, *i.e.*, $a<\text{ε}\sqrt{2bD}$, F is a monotone decreasing Function of a. Thus rule (iv) is obtained.

It is necessary to point out that the condition of F=1 is not sufficient for us to judge whether SR occurs unless other factors are under consideration in addition. However, the judgmental Equation (8) can explain the effect of each parameter on the occurrence of SR and the relationship between parameters. Therefore, as a qualitative-analysis tool, Equation (8) and the relevant four rules are helpful to theoretically analyze the GPASR mechanism in Duffing Equation (6).

As to a given test signal, A, $f_{0}$ and D are determinate and nonadjustable signal parameters. *k*, *a*, *b*, *ε* and *R* are the adjustable parameters, the values of which are always initialized in Equation (6) in advance. When the signal parameters do not match with the given Duffing system, namely F≠1, we should adjust one or more adjustable parameters to satisfy F=1, and the adjusted mechanism and rules can be obtained through analyzing Equation (8). This is the logic explanation for the GPASR algorithm. Next, we will study the mechanism of GPASR when the signal amplitude, frequency, and noise intensity of test signal do not match with the Duffing system.

### 3.3. GPASR in a Duffing Oscillator under Unmatched Signal Amplitude

Although the value of *F* is independent of *A* according to Equation (8), situations of unmatched signal amplitude still exist, which are mainly reflected in: (i) when $A>A_{C}$, the Brownian particle can transit only under the action of a periodic driving force. Noise does not play a positive role in the transition and is merely a perturbing disturbance. This situation does not belong to the definition of SR; (ii) when A is much too small, the Brownian particle can hardly transit in accordance with the signal characteristics even though noise exists; SR cannot take place either. Therefore, the premise for Duffing system to produce SR and remain independent of A is that A satisfies the small-parameter condition for the system to produce SR. 

**Figure 4:**
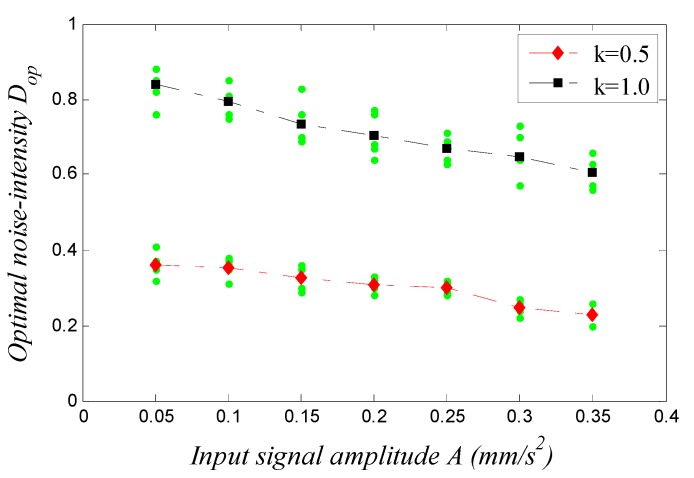
Dependence of the optimal noise-intensity $D_{op}$ for SR to occur in a Duffing system on the input signal amplitude A.

In Equation (6), both ε and R are set at 1, respectively, the damping ratio is set at either k=0.5 or k=1, and other parameters are set as the conditions (5). The dependence of the optimal noise-intensity $D_{op}$ for SR to occur on input signal amplitude A ($A<A_{C}$) can be plotted in Figure 4 (note that, to eliminate the influence of different white noise, we applied five different input Gaussian white noise samples for each A, obtained five corresponding $D_{op}$, and used the mean value to present the figure).

Given $A<A_{C}$, Figure 4 shows that the values of $D_{op}$ (i) remain in a relative stable range irrespective of A. That is to say, the value of A has little influence on that of $D_{op}$. This rule is in accord with the qualitative analysis in Section 3.2; (ii) present a slow decline as A increases, the reason being that the more A nears $A_{C}$, the less energy the Brownian particle needs to accomplish a transition, hence $D_{op}$ will decrease accordingly. In contrast, the farther A is from $A_{C}$, the more $D_{op}$ increases accordingly; and (iii) increase with the increase of k, which will be explained in Section 3.5.1. It can be seen that when the input signal amplitude is an appropriate small-parameter, parameters do not need to be adjusted because A has little influence on the occurrence of SR.

Thus when A does not match with other parameters, the rules for realizing GPASR in Duffing Equation (6) can be obtained. We only need to and only can adjust the value of ε to make the transformed input signal amplitude εA an appropriate small-parameter, *i.e.*, $\text{ε}A<A_{C}$. Obviously, there exists an adjustable range $\text{Ε}=({\text{ε}}_{\text{min}},{\text{ε}}_{\text{max}})$ for ε to adapt the value of εA. Equation (6) cannot produce SR when ε is too large ($\text{ε}>{\text{ε}}_{\text{max}}$) or too small ($\text{ε}<{\text{ε}}_{\text{min}}$).

### 3.4. GPASR in a Duffing Oscillator under Unmatched Signal Frequency

The characteristic frequency $f_{0}$ of a test signal is a significant parameter. A prerequisite for a Duffing system to produce SR is that $f_{0}$ must be a small-parameter satisfying the conditions of adiabatic approximation theory (as $f_{0}=0.01$ Hz in parameter condition (5)). However, the frequency of the actual test signal may be far beyond this small-parameter limit. With the increase of frequency $f_{0}$ the system response x(t) increasingly lags behind the input, which is manifested by $r_{K}\ll 2f_{0}$ or F≪1 from the perspective of Kramers rate. The transition of Brownian particle cannot follow the switching rate of periodic signal, so the system cannot produce SR. Therefore, the situations that signal frequency does not match with other parameters mainly indicate that signals are of large-frequency. The GPASR for large-frequency signals is studied in this section.

We also analyze this through Equation (8). A large frequency $f_{0}$ leads to F<1, so the adjustments of parameters should increase the value of F so that F=1 can be satisfied. It can be intuitively seen from the rules (ii) and (iii) in Section 3.2 that the increase of b, ε, R and the decrease of k can increase the value of F. However, Equation (8) tells that the exponential term on the right-hand side restrict the transition rate of Brownian particle, and then restrict the value of F. That is to say, no matter what the values of parameters a, b and ε are, the relation of $0<\text{exp}\left(-a^{2}/4b{\text{ε}}^{2}D\right)<1$ is always there; thus the restriction for F can be expressed as:

(10) $$F=\frac{aR}{2\sqrt{2}\text{π}kf_{0}}\text{exp}\left(-\frac{a^{2}}{4b{\text{ε}}^{2}D}\right)\in (0,\frac{aR}{2\sqrt{2}\text{π}kf_{0}})$$

We know from Equation (10) that, as to a group of certain parameters, if the signal frequency $f_{0}$ is large enough to make $aR/(2\sqrt{2}\text{π}kf_{0})<1$, the relation of F=1 (i) obviously, cannot be satisfied by adjusting the value of b or ε separately; (ii) can be satisfied by adjusting the value of k. With $f_{0}$ increasing to a large parameter, we can decrease k to an appropriate value thus satisfying F=1. However, if k is too small, the transition speed of Brownian particle does not follow the Kramers rate any more, it becomes improper to decide whether SR occurs by F=1, thus k only has limited adjustable range.

Moreover, the rule (iv) in Section 3.2 tells that the value of F(a) first increases and then falls as the value of a increases, the peak is at $a=\text{ε}\sqrt{2bD}$; that is:

(11) $$F(a)\leq \frac{\text{ε}\sqrt{2bD}R}{2\sqrt{2}\text{π}kf_{0}}\text{exp}\left(-\frac{{\text{ε}}^{2}2bD}{4b{\text{ε}}^{2}D}\right)=\frac{\text{ε}\sqrt{bD}R}{2\text{π}kf_{0}}\text{exp}\left(-\frac{1}{2}\right)$$

when $f_{0}$ is a large parameter:

(12) $$F(a)\leq \frac{\text{ε}\sqrt{bD}R}{2\text{π}kf_{0}}\text{exp}\left(-\frac{1}{2}\right)\ll 1$$

so it is impossible to satisfy the relation of F=1 and produce SR in the system by adjusting the value of a separately.

The above analyses imply that the only parameter we can adjust separately to realize the SR in a Duffing oscillator under large-frequency conditions is the scale-transformation coefficient R. The purpose of adjusting R is to transform the time/frequency scale for the test signals, with detailed meaning as follows [15,24]: as to a test signal frequency sampled using frequency $f_{s}$, which contains a signal component with large-frequency $f_{0}$, the time interval of the discrete data is $\Delta t=1/f_{s}$; input the test signal into the Duffing oscillator, then we introduce the scale-transformation coefficient R (>1), and numerically solve the equation using calculation step $\Delta t^{\prime }=R\times \Delta t=R/f_{s}$, thus rescaling the time interval of the signal by factor R and compressing the frequency of periodic signal by R, namely ${f^{\prime }}_{0}=f_{0}/R$. Thus, the original signal with frequency $f_{0}$ and sampling frequency $f_{s}$ becomes a new signal with frequency $f_{0}^{\prime }=f_{0}/R$ and sampling frequency $f_{s}^{\prime }=f_{s}/R$. Here, $f_{s}^{\prime }$ is called the scaled sampling frequency.

The rules (ii) and (iii) in Section 3.2 tell that there is a positive correlation between R and $f_{0}$ in guaranteeing the relation of F=1 when other parameters remain constant. That is to say, a large-frequency signal should match with a large R. We know from Equation (6) that the system can produce SR with the adjustment of R to make $f_{0}^{\prime }=f_{0}/R$ satisfy small-parameter limits.

### 3.5. GPASR in a Diffing Oscillator under Unmatched Noise-Intensity

The noise intensity has significant influence on the SR. On one hand, when noise-intensity is too small, the Brownian particle cannot accumulate enough energy to accomplish the transition, and the system output is under-resonant. On the other hand, when noise-intensity is too large and exceeds the level the system needs to produce SR, the unnecessary noise will be residual noise, which will inundate the characteristic signal of the system output. The system output under this scenario is over-resonant. Therefore, there exists an optimal noise-intensity for SR in a Duffing oscillator under a group of certain parameters, as $D_{op}$ in Figure 3. However, the noise-intensity of test signal seldom satisfies this condition, this is because on one hand the background noise is too large when compared with the weak signal components in test signal, and on the other hand the noise-intensity does not match with other parameters. Therefore, the GPASR in a Duffing oscillator under unmatched noise-intensity can meet two purposes: to realize SR under large-intensity noise conditions, and adjusting other parameters to match with the noise-intensity. The adjusted rules under unmatched noise-intensity have been studied in [31] in-depth. In this section, we will only give simple introductions and essential analyses.

#### 3.5.1. Adjustment of Damping Ratio *k*

There is a positive correlation between *k* and *D* in guaranteeing the relation of *F* = 1 when other parameters remain constant. That is to say, a large *k* should be chosen to match with a signal with large-intensity noise and vice versa; this is accordant with the rule (iii) of Figure 4 in Section 3.3. The relation of $0<\text{exp}\left(-a^{2}/4b{\text{ε}}^{2}D\right)<1$ is always satisfied in Equation (8), thus there always exists an appropriate *k* to make *F* = 1 no matter what value of *D* is. This implies that it is possible to realize SR for signals with any noise-intensity by adjusting the damping ratio: the value of *k* should be decreased under under-resonant conditions (*D* is a small parameter), or be increased under over-resonant conditions ( *D* is a large parameter).

#### 3.5.2. Adjustment of System Parameter *a*

The value of F(a) first increases and then falls as the value of *a* increases, the peak is at $a=\text{ε}\sqrt{2bD}$. According to in Equation (11), the following relation must be satisfied in order to meet the relation of F=1:

(13a) $$\frac{\text{ε}\sqrt{bD}R}{2\text{π}kf_{0}}\text{exp}\left(-\frac{1}{2}\right)\geq 1$$

(13b) $$D\geq \frac{4e{\text{π}}^{2}k^{2}f_{0}^{2}}{{\text{ε}}^{2}bR^{2}}$$

*i.e*., Define $D_{C}=(4e{\text{π}}^{2}k^{2}f_{0}^{2})/({\text{ε}}^{2}bR^{2})$. We see in Equation (13) that the adjustment of a has limited influence on realizing SR for signal with small noise intensity. That is to say, if $D<D_{C}$, the system cannot produce SR by adjusting the value of *a* only. The value of $D_{C}$ depends on the coaction of *k*, *b*, *ε* and *R*.

Under the condition of $D>D_{C}$, there is first a negative correlation and then a positive correlation between a and D in guaranteeing the relation of F=1 when other parameters remain constant. From this perspective, as to signals with large noise-intensity, a small (when $a<\text{ε}\sqrt{2bD}$) or a large (when $a>\text{ε}\sqrt{2bD}$) value of a should be chosen; but as to signals with small noise-intensity, a parameter a whose value is close to $\text{ε}\sqrt{2bD}$ should be chosen.

However, as the critical value $\text{ε}\sqrt{2bD}$ for parameter a to maximize the value of F changes as D changes, the rule for adjusting *a* becomes uncertain. Only some qualitative results can be obtained in studying the adjustment rules of *a*; no general adjustment rules exist.

#### 3.5.3. Adjustment of System Parameter *b*

There is a negative correlation between b and D in guaranteeing the relation of F=1 when other parameters remain constant. That is to say, a small b should be chosen to match with a signal with large-intensity noise and *vice versa*. We know from Equation (8) that there always exists an appropriate b to make F=1 no matter what value of D is. This implies that it is possible to realize SR for signals with any noise-intensity by adjusting b: the value of b should be increased under under-resonant conditions, or be decreased under over-resonant conditions.

#### 3.5.4. Adjustment of Amplitude-Transformation Coefficient ε

There is a negative correlation between ε and D in guaranteeing the relation of F=1 when other parameters remain constant, and there always exists an appropriate ε to make F=1 no matter what the value of D is. It is possible to realize SR for signals with any noise-intensity by adjusting the amplitude-transformation coefficient ε and the adjustment rules is the same with that of b: the value of ε should be increased under under-resonant conditions, or be decreased under over-resonant conditions.

Of special note is that although there always exists an appropriate ε to match with any value of D according to judgmental Equation (8), ε has a limited adjustable range. We know from Section 3.3 that the value of input signal amplitude is also transformed following the adjustment of ε, there exists an adjustable range $\text{Ε}=({\text{ε}}_{\text{min}},{\text{ε}}_{\text{max}})$ for ε to make the input signal amplitude εA an appropriate small-amplitude that satisfies $\text{ε}A<A_{C}$. This implies that the value of ε can only be chosen among this range to realize SR in a Duffing oscillator under unmatched noise-intensity.

#### 3.5.5. Adjustment of Scale-Transformation Coefficient *R*

There is a negative correlation between R and D in guaranteeing the relation of F=1 when other parameters remain constant. The adjustment rules of R is the same with that of b and ε: the value of R should be increased under under-resonant conditions, or be decreased under over-resonant conditions. 

Of special note is that if the D is too large, R need to be decreased to a small value. So the increasing input signal frequency $f_{0}^{\prime }=f_{0}/R$ will easily exceed the appropriate small-parameter limit thus deteriorating the SR output result; if D is too small, a large R should be chosen to match with it, thus producing a small scaled sampling frequency $f_{s}^{\prime }=f_{s}/R$; the calculation step $\Delta t^{\prime }=1/f_{s}^{\prime }=R/f_{s}$ will be too large and result in calculation error or even result in overflow. Therefore, R can only be adjusted among a certain range to realize SR in a Duffing oscillator under unmatched noise-intensity.

### 3.6. Conclusion of the GPASR Rules of a Duffing Oscillator

Table 1 gives a summary of GPASR rules in a Duffing oscillator under unmatched signal amplitude *A*, frequency $f_{0}$ (being large-parameter) and/or noise-intensity *D*. When the input signal amplitude does not match with the system, ε is the only parameter we can adjust to realize the SR in Duffing oscillator; when the input signal frequency is a large-parameter, we can only adjust *R* to realize the SR in a Duffing oscillator; while when the input noise-intensity does not match with the system, based on whether the system output is under-resonant or over resonant, the adjustments of *k*, *a*, *b*, ε and *R* are all helpful to realize the SR in a Duffing oscillator. Under this scenario, the adjustments of *k* and *b* are convenient; *a* can only be adjusted when $D>D_{C}$ and has a complicated adjustment rule; ε and *R* can only be adjusted among a certain range.

**Table 1:** GPASR rules in a Duffing oscillator under unmatched signal amplitude *A*, frequency *f*_0_ (being large-parameter) and/or noise-intensity *D*.

		*k*	*a*	*b*	ε	*R*
A	Large	Invalid	Invalid	Invalid	Decrease ε	Invalid
	Small	Invalid	Invalid	Invalid	Increase ε	Invalid
*f*_0_ is a large-parameter		Invalid	Invalid	Invalid	Invalid	Adjust *R* to make $f_{0}/R$ satisfy the small-parameter limits
D	Large	Increase *k*	Adjust *a* far from $\text{ε}\sqrt{2bD}$ when $D>D_{C}$	Decrease *b*	Decrease ε appropriately	Decrease *R* appropriately
	Small	Decrease *k*	Adjust *a* near $\text{ε}\sqrt{2bD}$ when $D>D_{C}$	Increase *b*	Increase ε appropriately	Increase *R* appropriately

## 4. Engineering Applications

### 4.1. Weak-signal Detection Method Based on GPASR of a Duffing Oscillator

According to the summative rules, we obtain the basic ideas of GPASR under unmatched signal and/or noise. First we adjust ε and R respectively until the input scaled signal amplitude and frequency satisfy small-parameter limits; and then adjust *k*, *a* and *b* to match with the noise-intensity. SR in Duffing oscillator appears under appropriate parameters.

**Figure 5:**
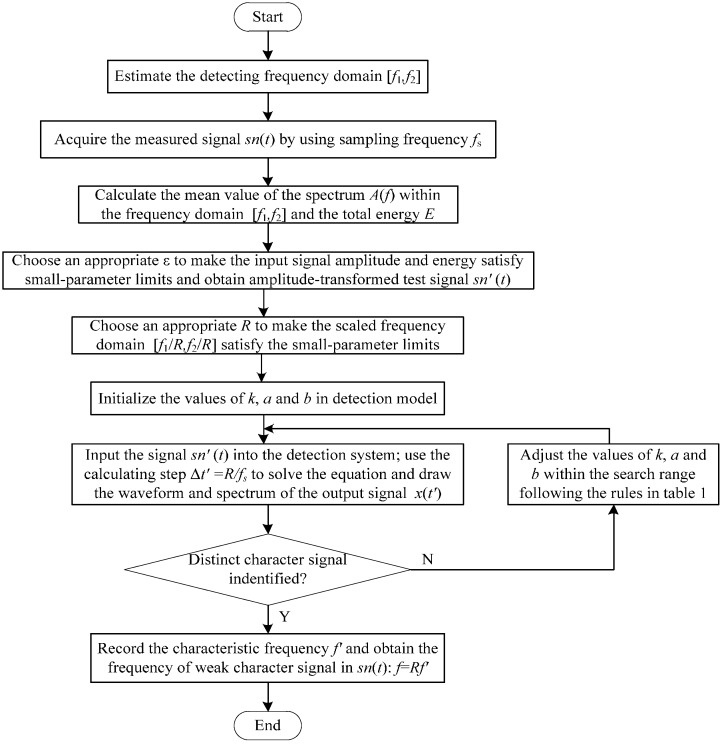
The flowchart of the proposed GPASR method.

The weak-signal detection model based on GPASR of a Duffing oscillator is:

(14) $$\ddot{x}(t^{\prime })+k\dot{x}(t^{\prime })-ax(t^{\prime })+bx^{3}(t^{\prime })=sn^{\prime }(t^{\prime })$$

whose parameters have the same means with Equation (6). Here, $sn^{\prime }(t^{\prime })=\text{ε}sn(t^{\prime })$ is the processed signal of the original test signal after an amplitude-transformation and a scale-transformation; $t^{\prime }$ is the time scale transformed by R. The flowchart of the GPASR method to identify the frequency of weak characteristic signal is shown in Figure 5.

We conducted a large number of calculations to select the empirical values and used fault diagnostics of the mechanical equipment to exemplify details of the detection procedure:

Step 1: Pre-analyze possible faults of the operating equipment, and estimate the detecting frequency domain $\left[f_{1},f_{2}\right]$ of the weak fault character signal according to the fault mechanism. Collect the vibration of the operating equipment with appropriate sampling frequency $f_{s}$ then acquire the measured signal sn(t);

Step 2: Get the amplitude spectrum sn(f) of the test signal sn(t) by fast Fourier transform (FFT) analysis. Calculate the mean value of the spectrum A(f) within the frequency domain $\left[f_{1},f_{2}\right]$, *i.e.*, A¯=1M∑f1≤f≤f2A(f), where M is the number of spectral lines within the frequency domain. Calculate the total energy E of the test signal. Multiply sn(t) by an appropriate ε, with the magnitude of $\text{ε}\bar{A}$ within 10^−3^–10^−1^ and that of ${\text{ε}}^{2}E$ within 10^−1^–10^1^, to make the input signal amplitude $\text{ε}\bar{A}$ and energy ${\text{ε}}^{2}E$ satisfy small-parameter limits. The amplitude-transformed test signal is denoted as $sn^{\prime }(t)$;

Step 3: Introduce an appropriate R to make the scaled frequency domain $\left[f_{1}/R,f_{2}/R\right]$ of the scale-transformed signal satisfy the small-parameter limits. Set the initial values of k, a and b in Equation (14). Input the signal $sn^{\prime }(t)$ into the detection system, and use the calculating step $\Delta t^{\prime }=1/f_{s}^{\prime }=R/f_{s}$ to solve Equation (14), thus the signal $sn^{\prime }(t)$ scale-transforms to $sn^{\prime }(t^{\prime })$, and the scaled sampling frequency $f_{s}^{\prime }=f_{s}/R$;

Step 4: Draw the waveform and spectrum of the output signal $x(t^{\prime })$. If we can identify a distinct character signal from the spectrum, record its frequency $f^{\prime }$; otherwise, adjust the value of k, a and b following the rules in Table 1 in accordance with the output state of the system until we can identify a distinct character signal from the spectrum. If no distinctive character signal is found within the search range, no fault characteristic signal exists;

Step 5: Obtain the frequency of the weak character signal in the test signal sn(t) in accordance with the result of Step 4, $f=Rf^{\prime }$, or prove that no weak character signal components exist; then assess and identify the fault of operating equipment accordingly.

### 4.2. Practical Examples

#### 4.2.1. Diagnosis of a Rotor Shaft-Bending Fault

Shaft bending, a common type of fault in rotating machinery, refers to a situation when the axis of shaft does not overlap with that of the rotation, resulting in vibrations in the unbalanced rotor. The vibration signal of the rotator has an obvious fundamental frequency, often accompanied by second or higher harmonic frequency components.

**Figure 6:**
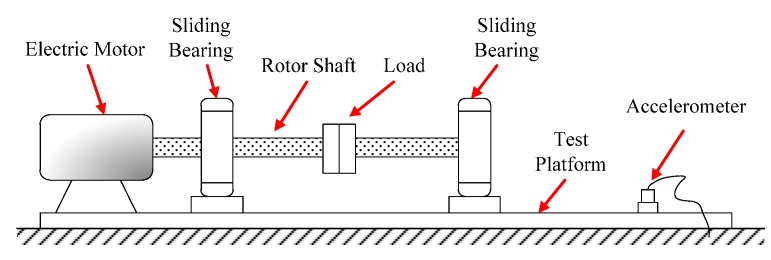
Sliding-bearing experimental table for shaft-bending fault experiments.

Shaft-bending fault experiments were performed on a sliding-bearing experimental table shown in Figure 6. The diameter of the shaft was ϕ12 mm. With a deviation of 0.38 mm between the shaft axis and the rotation axis, shaft-bending faults were present in the sliding-bearing rotor system. To simulate weak fault conditions, an accelerometer was installed on the experimental table 0.5 meters far from the bearing base. The vibration signal of the shaft-bending fault would thus be damped because of the bearing and experiment structure, and the sensor would then be able to record the vibration signal of the weak fault.

**Figure 7:**
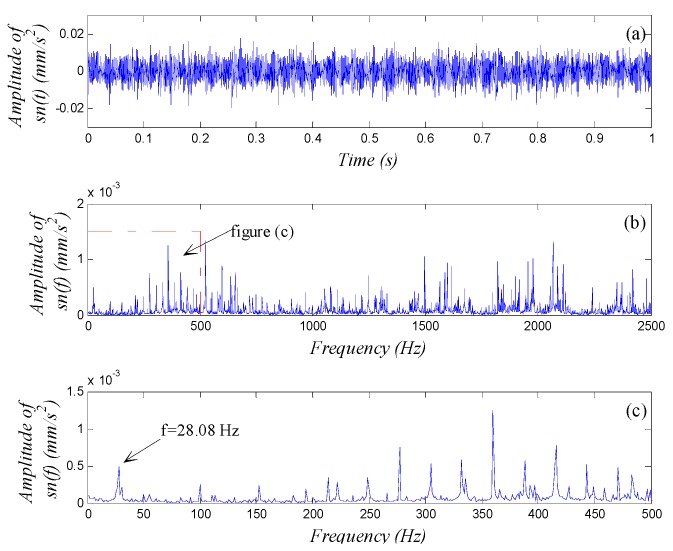
Vibration signal analysis of shaft-bending fault: (**a**) waveform; (**b**) global spectrum; (**c**) low-frequency spectrum.

The motor-driven rotor was rotated at an angular speed of 1680 rpm, *i.e.*, a frequency of 28 Hz. An NI PXI-1033, a signal-acquiring device developed by National Instrument Corporation (Austin, TX, USA) collected the data from the accelerometer at a sampling frequency of $f_{s}=5000$ Hz based on the detecting frequency domain [20 Hz, 35 Hz] and sampling length of N=5000. Calculations for 4096 points were made to obtain the waveform, global spectrum and low-frequency spectrum (0–500 Hz, averaged over 10 cycles as the global spectrum); see Figure 7.

We see from Figure 7 that from the accelerometer far from the vibration source, the fault signal was weak, nearly being immersed in background noise during wave propagation. In the spectra in Figure 7b,c, we can see the signal component with frequency f=28.08 Hz, which is equal to the rotational frequency. However, its spectral line was not as prominent when compared with other spectral lines; we could not observe any obvious multiple-frequency components either. So it was impossible to decide whether the rotor had a fault and what the fault was if any. To further diagnose whether the rotor had a fault, we employed the weak-signal detection method based on GPASR of Duffing oscillator proposed in this paper to analyze the signal following the detection procedure detailed in Section 4.1.

First, we select an appropriate value for ε. According to the spectrum in Figure 7b, we obtained the mean value of the spectrum A(f) within the detecting frequency domain, *i.e.*, $\bar{A}=1.635\times 10^{-4}$, and the total energy $E=2.4292\times 10^{-5}$ of the test signal. ε was set at 200 preliminarily, thus $\text{ε}\bar{A}=0.0327$ and ${\text{ε}}^{2}E=0.97$ both satisfied the small-parameter limits. The amplitude-transformed test signal was denoted as $sn^{\prime }(t)$;

Second, we select an appropriate value for R. According to the detecting frequency domain and the value of sampling frequency, we set R=1000 preliminarily so that the scaled detecting frequency domain [0.02 Hz, 0.035 Hz] of the transformed character signal satisfied the small-parameter limits.

**Figure 8:**
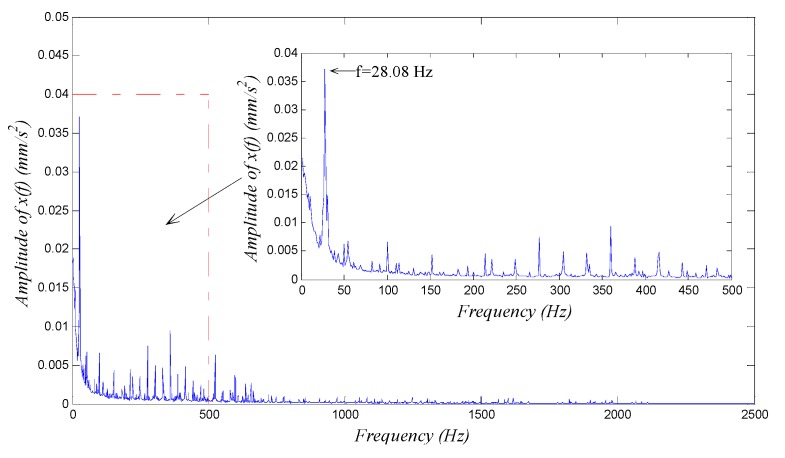
The output global and low-frequency spectrums of generalized parameter-adjusted Duffing system with the vibration signal of sliding-bearing experimental table input. The corresponding parameters in the detection system are set to ε=200, R=1000, a=b=1, and k=11.

Next, we set initial values of k=a=b=1 preliminarily in the detection system Equation (14) and input the signal $sn^{\prime }(t)$. We set the calculating step $\Delta t^{\prime }=1/f_{s}^{\prime }=1/5$ s to solve Equation (14), thus the signal $sn^{\prime }(t)$ was scale-transformed to $sn^{\prime }(t^{\prime })$, and the scaled sampling frequency $f_{s}^{\prime }=5$ Hz;

Last, we chose k as main adjusted parameter and drew the waveform and spectrum of the output signal $x(t^{\prime })$. We adjusted the value of k following the rules of Table 1 in accordance with the distribution shape of the spectrum until we could identify a distinct character signal from the spectrum. The output signal spectral line was most prominent when k=11. The detection result is shown in Figure 8 with the frequency parameter being scale-transformed using the real sampling frequency.

We see from Figure 8 that the output spectrum exhibits a signal component with amplitude much larger than others, whose frequency f=28.08 Hz, and no multiple-frequency signal components are observed. This result shows that the character signal of rotational frequency is the most prominent signal component of the test signal; this situation infers that there must be a shaft-bending fault or a misalignment fault in the rotor. The most distinct difference between the two faults is that obvious second or higher harmonic frequency components exist in the vibration signal of the rotor with a shaft-bending fault, while does not exist in that with a misalignment fault.

**Figure 9:**
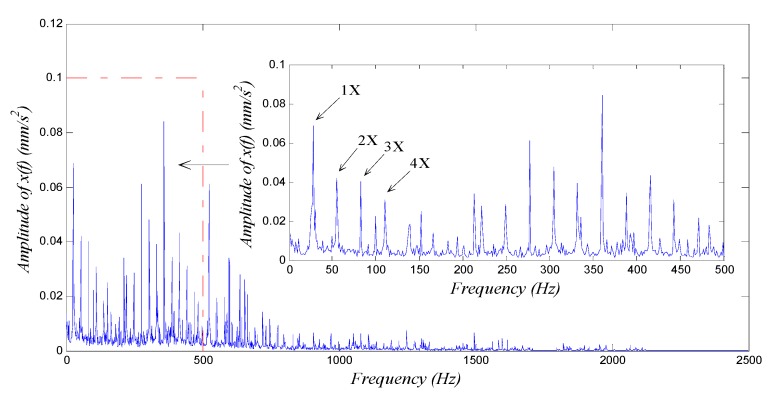
The output global and low-frequency spectra of a generalized parameter-adjusted Duffing system with the vibration signal of sliding-bearing experimental table input. The corresponding parameters in the detection system are set to ε=200, R=2500, a=b=1, and k=3.

In order to further confirm what kind of fault the rotor had, we continued to adjust the parameters of the detection system Equation (14). Because the potential second or higher harmonic frequencies were larger than the fundamental frequency, we should increase the value of R according to Table 1. We set R=2500 preliminarily and adjusted the value of k; when k=3, the output spectrum was shown in Figure 9, in which there were obvious fundamental frequency component and also the second and higher harmonic frequency components. Thus we were able to tell there was probably a shaft-bending in the rotor system, which was accordant with the real physical truth of the system. Thus the diagnosis of a shaft-bending fault was realized by using the GPASR method of a Duffing oscillator.

#### 4.2.2. Diagnosis of a Rolling Bearing Outer Ring Fault

**Figure 10:**
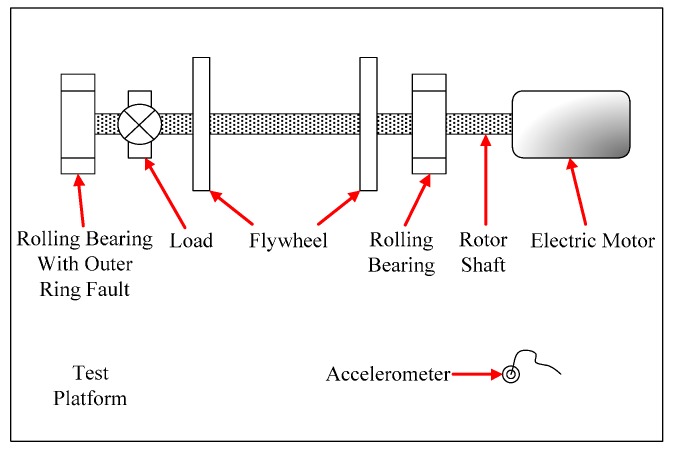
Rolling bearing fault experimental table.

The rolling bearing, an element widely used in mechanical equipment, consists of an outer ring, an inner ring, rolling elements and a cage. When a fault caused by local damage occurs in the surface of an element of the rolling bearing, the surface fault will periodically strike the surface of other elements as the bearing rotates. This produces a uniformly spaced pulsed force, whose impacting frequency is decided by aspects such as the geometry of the bearing, the rotating speed of shaft, and the position of the fault. When the fault forms on the surface of the bearing’s outer ring, the characteristic frequency of the fault is:

(15) $$f_{out}=\frac{z}{2}(1-\frac{d}{D}\text{cosβ})f_{0}$$

where z is the number of rolling elements, β the contact angle, d the diameter of rolling element, D the pitch diameter of rolling element, and $f_{0}$ the rotating frequency of bearing. Research results show that when the outer ring of rolling bearing bears the fault, there exist regular spectral peaks associated with the fault characteristic frequency $f_{out}$ and its higher harmonics [36]. However, the vibration signal of such faults is always modulated, containing a high-frequency meshing carrier and a low-frequency impulse modulation wave. Hence it is difficult to identify the low-frequency characteristic signal of the fault in the spectrum through analyzing the modulated vibration signal directly. In particular, if the impulse signal is not obvious resulting from an early weak fault in the rolling-bearing outer ring or a large-intensity noise background, it is hard to extract the fundamental frequency of the fault signal from the spectrum of the modulated signal. Experiments were performed on the rolling-bearing experimental table shown in Figure 10. The type of ball bearing used in the experiment was the NU205, with specifications z=13, d=7.5 mm, D=39 mm, and β=0. A 0.2-mm-wide, 0.1-mm-deep groove was wire-cut on the surface of the outer ring to mimic a bearing fault. To simulate early weak fault conditions, we installed an accelerometer on the experimental table 0.8 meters far from the fault bearing. Thus the vibration signal is dampened because of the long propagation distance, and the sensor would then be able to record the vibration signal of the weak fault.

**Figure 11:**
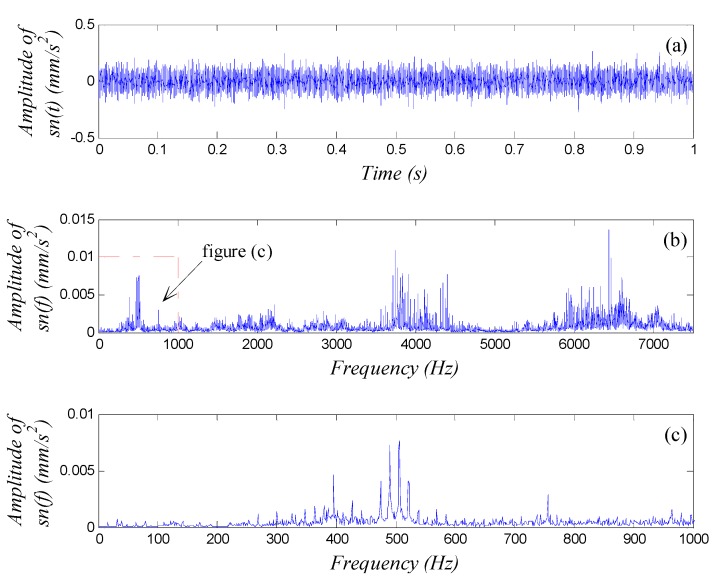
Vibration signal analysis of an outer ring fault in a rolling bearing: (**a**) waveform; (**b**) global spectrum; (**c**) low-frequency spectrum.

Assume that we already know the rolling bearing had a fault, but we didn’t know where the fault was. We tried to analyze the vibration signal of the system and find out the fault type. Take the outer ring fault diagnosis as example. The observable signal components with characteristic frequency $f_{out}$ and its higher harmonics in the vibration fault of the system would be sufficient to confirm an outer ring fault. The motor-driven rotor rotated with a speed of 950 rpm ($f_{0}=15.8$ Hz), thus if an outer ring fault exists, a characteristic frequency for the fault would be $f_{out}=82.95$ Hz. The NI PXI-1033 was used again to collect data from the accelerometer at a sampling frequency of $f_{s}=15,000$ Hz according to the possible fault characteristic frequency domain [75 Hz, 90 Hz] and sampling length of *N* = 15,000. An analysis of 12,288 points was performed to obtain the waveform, global spectrum and low-frequency spectrum (0–1000 Hz, averaged over 10 cycles as global spectrum) shown in Figure 11.

We did not observe any characteristic features of the fault in the signal waveform Figure 11a or the spectra in Figure 11b,c, so we cannot say whether the bearing had an outer ring fault. Again, we employed the weak-signal detection method based on GPASR of a Duffing oscillator to analyze the signal following the detection procedure detailed in Section 4.1, and further diagnosed whether the bearing had an outer ring fault.

First, we select an appropriate value for ε. According to the spectrum in Figure 11b, we obtained the mean value of the spectrum A(f) within the detecting frequency domain, *i.e.*, $\bar{A}=1.33\times 10^{-4}$, and the total energy E=0.0036 of the test signal. The amplitude-transformation coefficient was set at ε=100 preliminarily, thus $\text{ε}\bar{A}=0.013$ and ${\text{ε}}^{2}E=36$ both satisfied the small-parameter limits. The amplitude-transformed test signal was denoted as $sn^{\prime }(t)$;

Second, we select an appropriate value for R. According to the detecting frequency domain and the value of sampling frequency, we set R=5000 preliminarily so that the scaled detecting frequency domain [0.015 Hz, 0.018 Hz] of the transformed character signal satisfied the small-parameter limits.

Next, we set initial values of k=8 and a=b=1 preliminarily in the detection system Equation (14) and input the signal $sn^{\prime }(t)$. We set the calculating step $\Delta t^{\prime }=1/f_{s}^{\prime }=1/3$ s to solve Equation (14), thus the signal $sn^{\prime }(t)$ was scale-transformed to $sn^{\prime }(t^{\prime })$, and the scaled sampling frequency $f_{s}^{\prime }=3$ Hz.

**Figure 12:**
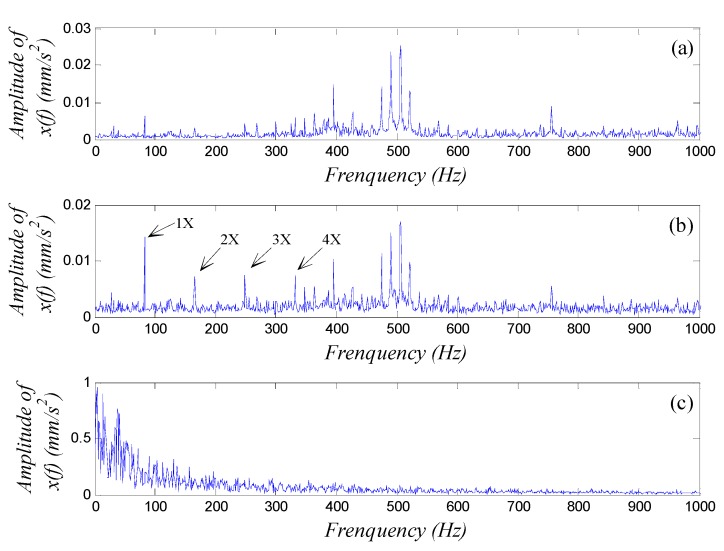
The output low-frequency spectra of a generalized parameter-adjusted Duffing system with the vibration signal of rolling-bearing experimental table input. The corresponding parameters in the detection system are set to ε = 100, *R* = 5000, a = b = 1, and (**a**) *k* = 9.5; (**b**) *k* = 10; (**c**) *k* = 10.5.

Last, we chose k as main adjusted parameter and drew the waveform and spectrum of the output signal $x(t^{\prime })$. We adjusted the value of k following the rules of Table 1 in accordance with the distribution shape of the spectrum until we could identify a distinct character signal from the spectrum. The best detection result with k=10 was shown in Figure 12b with the frequency parameter scale-transformed using the real sampling frequency. We can see the fundamental frequency at f=83.1 Hz and also the harmonics from n = 2–4 in the SR output low-frequency spectrum, which confirmed the presence of a fault in the outer ring. 

For comparison, Figure 12a shows the output low-frequency spectrum with *k* = 9.5, the amplitude at characteristic frequency is weak and the system is still over-resonant, and the SR output can be realized by increasing the value of k; and Figure 12c shows the output low-frequency spectrum with *k* = 10.5, low-frequency disturbance is strong and the system is under-resonant, and the SR output can be realized by decreasing the value of *k*; these results are accordant with the analysis of Section 3.5.1. The diagnosis of an outer ring fault was realized by using the GPASR method of a Duffing oscillator. Similarly, if we want to confirm whether a fault exists in the inner ring, rolling elements or cage, we need to compute the corresponding characteristic frequency and collect the vibration signal of the system at an appropriate sampling frequency, and then analyze the signal by using the proposed weak-signal detection method. A corresponding fault can be diagnosed according to the GPASR output.

### 4.3. Discussion

The frequency-domain analysis method based on FFT is the basis of modern signal processing. However, the weak periodic signal submerged in strong background noise, such as the incipient fault signal of mechanical equipment, cannot be identified by a simple FFT method, as shown in Figure 7 and Figure 11. Obviously, the detection results of the two practical examples in Section 4.2 present the superiority of SR of a Duffing oscillator in detecting weak periodic signals.

The biggest difficulty in applying SR in practical engineering is the complexity of parameter adjustments. Many adaptive optimization algorithms have been proposed to obtain optimal parameters for an one-dimensional Langevin system to produce SR, while few researches focus on the parameter-adjusted methods for a two-dimensional Duffing system. The GPASR method proposed in this paper completely reveals the parameter-adjusted mechanism and rules for a Duffing oscillator to produce SR. Compared to the adaptive optimization algorithms of a Langevin system, this method has two advantages: (i) the tunable damping ratio in the two-dimensional model makes the system more adaptive to signals of different noise intensity; we can only adjust the damping ratio in a Duffing system to match with the noise intensity while we must adjust several parameters simultaneously in a Langevin system; (ii) we adjust the parameters basing on a comprehensive understanding of SR mechanism instead of passively accepting the optimization results, thus we can easily confirm whether the detection result is optimal.

The two practical examples also demonstrate the feasibility of the proposed approach in practical engineering application. We can acquire optimal output following the detection procedure and identify the characteristic frequency of fault signal. The proposed method is also effective in detecting modulated weak signal. However, the successful application of this method relies on the rich experience of the experimenter in recognizing the output state of system. It is not so convenient when compared with the adaptive optimization algorithms, and SR output may not been obtained by an inexperienced experimenter even though the weak characteristic signal exists.

## 5. Conclusions and Summary

SR of a two-dimensional Duffing oscillator describes an optimal matching relation of signal, noise and nonlinear system. The biggest difficulty of the application of a Duffing oscillator as a weak-signal detector is that the signal amplitude, frequency and/or noise-intensity of the test signal do not always optimally match with the nonlinear system. A GPASR model of a Duffing oscillator was presented in this paper, whose parameters contain not only signal parameters, noise intensity and system parameters, but also the amplitude-transformation coefficient and time/frequency scale-transformation coefficient used in practical engineering application. A judgmental Function for judging the occurrence of SR in generalized parameter-adjusted Duffing system was established based on Kramers rate and was used to analyze the parameters. Furthermore, we studied the mechanism of GPASR of a Duffing oscillator when the signal amplitude, frequency, and noise intensity of test signal are unmatched and obtained the adjusted rules: when the input signal amplitude does not match with the system, the amplitude-transformation coefficient ε is the only parameter we can adjust to realize the SR in a Duffing oscillator; when the input signal frequency is a large-parameter, we can only adjust the scale-transformation *R* to realize the SR in a Duffing oscillator; while when the input noise-intensity does not match with the system, the adjustments of *k*, *a*, *b*, ε and *R* are all helpful to realize the SR in a Duffing oscillator, under this scenario, the adjustments of *k* and *b* are convenient, *a* can only be adjusted when $D>D_{C}$ and has a complicated adjustment rule, ε and *R* can only be adjusted among a certain range. A weak-signal detection approach based on the GPASR of a Duffing oscillator was proposed at last. Last but not least, this paper gave detailed descriptions of the detection procedure and two practical examples to demonstrate the applicability of the proposed method.
